# Increased Plasma Lipocalin-2 Levels in Patients with Myelin Oligodendrocyte Glycoprotein-IgG–Positive Optic Neuritis

**DOI:** 10.3390/jcm11092635

**Published:** 2022-05-07

**Authors:** Jong-Heon Kim, Hyejin Lee, Junho Oh, Kyoungho Suk, Bo Young Chun

**Affiliations:** 1 Brain Science & Engineering Institute, School of Medicine, Kyungpook National University, Daegu 41944, Korea; jongheonkim.phd@gmail.com (J.-H.K.); ksuk@knu.ac.kr (K.S.); 2 Department of Ophthalmology, School of Medicine, Kyungpook National University, Daegu 41944, Korea; hjllls@naver.com (H.L.); justdoita@nate.com (J.O.); 3 Department of Pharmacology, School of Medicine, Kyungpook National University, Daegu 41944, Korea

**Keywords:** lipocalin-2, myelin oligodendrocyte glycoprotein IgG, optic neuritis, recurrence

## Abstract

This study aimed to evaluate the correlation between plasma lipocalin-2 (LCN2) levels and myelin oligodendrocyte glycoprotein (MOG)-immunoglobulin G (IgG) seropositivity in patients with optic neuritis. Peripheral blood samples were collected from 19 patients with optic neuritis and 20 healthy controls. Plasma LCN2 and MOG-IgG levels were measured using enzyme-linked immunosorbent assay and a cell-based assay, respectively. The correlation between plasma LCN2 levels and MOG-IgG titers in patients with optic neuritis was analyzed. Receiver operating characteristic (ROC) curves were constructed to assess and compare the ability of plasma LCN2 and MOG-IgG levels for predicting optic neuritis recurrence. Patients with MOG-IgG–positive optic neuritis had significantly higher mean plasma LCN2 levels than controls and patients with MOG-IgG–negative optic neuritis (*p* = 0.037). Plasma LCN2 and MOG-IgG levels were significantly correlated in patients with optic neuritis (*r* = 0.553, *p* = 0.0141). There were no significant differences in the areas under the ROC curve (AUC) of plasma LCN2 (0.693, 95% confidence interval [CI] 0.443–0.880, *p* = 0.133) and MOG-IgG (0.641, 95% CI, 0.400–0.840, *p* = 0.298) levels (95% CI, −0.266–0.448, *p* = 0.618). Plasma LCN2 levels may aid differentiation of MOG-IgG–positive optic neuritis from MOG-IgG–negative optic neuritis.

## 1. Introduction

Discovery of a novel glial autoantibody specific for myelin oligodendrocyte glycoprotein (MOG) has enabled recognition of antigen-specific inflammatory demyelinating diseases of the central nervous system (CNS) manifesting as optic neuritis [1,2,3]. MOG is expressed on the surface of myelin sheaths and oligodendrocytes in the CNS and is highly immunogenic in rodents and humans [4,5,6,7]. Studies on experimental autoimmune optic neuritis (EAON) have shown that optic neuritis can be induced by adjuvant immunization with MOG peptides [4,5,6]. The pathophysiologic role of MOG autoantibodies is not yet fully understood; however, MOG-specific B cells and MOG-immunoglobulin G (IgG) activate MOG-specific effector T cells via antigen-presenting cells [4,8,9]. The histopathologic characteristics of MOG-IgG lesions include demyelination with marked inflammatory cell infiltration, IgG and complement deposition, and relative preservation of axons and astrocytes [4,5,6,7,10].

MOG-IgG–positive optic neuritis is a recently discovered cause of optic neuropathy. Its pathophysiological and clinical characteristics differ from those of optic neuritis associated with multiple sclerosis or neuromyelitis optica [4]. Patients with MOG-IgG–positive optic neuritis present with initially severe vision loss, are likely to have optic nerve head edema, and have favorable visual outcomes [3,4,11]. Recurrences of short duration between optic neuritis attacks occur more frequently in patients with MOG-IgG–positive optic neuritis than in those with MOG-IgG–negative optic neuritis [3,4,11]. Therefore, close monitoring for signs of recurrence and long-term prophylactic immunosuppression may be necessary in patients with MOG-IgG–positive optic neuritis [3,4,11]. MOG-IgG titers are measured using cell-based assays, which have higher sensitivity and specificity than Western blot or enzyme-linked immunosorbent assay (ELISA); however, they are more expensive than Western blot or ELISA and require special laboratory equipment.

We previously reported that lipocalin-2 (LCN2) expression was highly upregulated in the optic nerve after EAON induction through MOG immunization [12]. LCN2, a secreted glycoprotein, has been found in plasma, urine, and cerebrospinal fluid [13,14,15]. LCN2 expression has been observed in reactive astrocytes after lipopolysaccharide administration and in patients with autoimmune encephalitis and other inflammatory CNS conditions [12,16,17]. LCN2 encodes proteins involved in the acute response to neuroinflammation, and it may amplify inflammation in the CNS by recruiting additional inflammatory cells [12,18]. We also demonstrated that patients with optic neuritis had significantly higher plasma LCN2 levels than healthy controls [12].

Therefore, we hypothesized that measuring the plasma LCN2 levels in patients with optic neuritis may help in the diagnosis of MOG-IgG–positive optic neuritis. We believe that the correlation of plasma LCN2 levels with MOG-IgG titers in patients with demyelinating optic neuritis has not been previously reported. The purpose of this study was to measure plasma LCN2 levels and evaluate their correlation with MOG-IgG titers in patients with optic neuritis.

## 2. Materials and Methods

### 2.1. Participants

This study was conducted from March 2018 to December 2020 at the Department of Ophthalmology, Kyungpook National University Hospital. The study was approved by the Institutional Review Board of Kyungpook National University Hospital and was conducted in accordance with the Declaration of Helsinki. Informed consent was obtained from all subjects. Out of 28 consecutive patients with demyelinating optic neuritis identified, 19 were included in this study. Patients with multiple sclerosis, neuromyelitis optica, or symptomatic CNS lesions other than those of the optic nerve; patients with incomplete clinical data; and patients who were followed up for less than 1 year were excluded. All patients had no history of neurological disease or new neurologic symptoms associated with their visual disturbance. As controls, we included 20 age- and sex-matched healthy individuals who had no conditions that could affect plasma levels of LCN2, such as infection, inflammatory disease, diabetes, or cancer, and had best-corrected visual acuities of 20/20. Optic neuritis was diagnosed based on the presence of acute visual symptoms, such as acute vision loss or visual field defects indicative of optic neuropathy with a relative afferent pupillary defect in the affected eye [3]. A Snellen chart was used to measure visual acuity, which was converted to logarithm of the minimum angle of resolution (LogMAR) visual acuity for statistical analysis.

### 2.2. Plasma LCN2 Level Measurement Using ELISA

LCN2 levels were measured in the plasma samples of the patients with optic neuritis and controls. Blood samples were collected on the day of optic neuritis diagnosis. High-dose intravenous steroid therapy was started after blood sample collection. Plasma LCN2 levels were measured using a commercially available Sandwich ELISA DuoSet kit (R&D systems, Minneapolis, MN, USA) according to the manufacturer’s instructions. The assays were performed in 96-well plates using 100 μL of plasma (1:400 dilution) per the manufacturer’s instructions. For standardization, human recombinant LCN2 protein was used at concentrations ranging from 39.06 to 2500 pg/mL. The results were normalized to the total protein content of the plasma samples. All measurements were determined from duplicated assays and averaged the results of the two assays.

### 2.3. Generation of MOG-Human Embryonic Kidney 293 Cells

Full-length human MOG (FL-MOG) cDNA was cloned into the KpnI and XbalI sites of the HG10364-UT pCMV3-MOG vector (Sino biological Inc., Beijing, China). All sequences were confirmed using a BigDye™ Terminator v3.1 cycle sequencing kit (Thermo Fisher Scientific, 4337455, Waltham, MA, USA) and an Applied Biosystems 3730 DNA analyzer (Thermo Fisher Scientific, Waltham, MA, USA). The cloned FL-MOG plasmid or the empty vector plasmid was transfected into human embryonic kidney 293 (HEK293) cells (ATCC, Manassas, VA, USA) using Effectene transfection reagent (Qiagen, 301425, Germantown, MD, USA). After 48 h, the cells were transferred into medium containing 100 μg/mL hygromycin (Thermo Fisher Scientific, Waltham, MA, USA). The hygromycin-resistant cells were isolated after 2 weeks and further cloned by limiting dilution. FL-MOG expression was confirmed through real-time polymerase chain reaction with the primer sets T7: 5′-TAATACGACTCACTATAGGG-3′ and BGH: 5′-TAGAAGGCACAGTCGAGG-3′.

### 2.4. Cell-Based Assay for the Detection of Serum Antibodies to the MOG Cell Surface

To detect the binding of patient serum IgG to surface MOG transduced in HEK293 cells, we conducted fluorescence-activated cell sorting (FACS) analysis as previously described [19]. Briefly, the transfected HEK293T cells were trypsinized and resuspended in Dulbecco’s modified Eagle’s medium, 2% fetal bovine serum, 1 mM ethylenediaminetetraacetic acid (FACS buffer) at 1.0 × 10^6^ cells/mL. The cells were centrifuged at 4 °C for 1 h. All further steps were carried out at 4 °C. Patient serum (diluted 1:50 in FACS buffer) was mixed with 1.0 × 10^5^ cells (100 µL). After rocking for 1 h, the cells were washed, and bound IgG was detected using Alexa Fluor 647 mouse anti-human IgG1 Fc antibody (5 μg/mL, A-10631, Thermo Fisher Scientific, Waltham, MA, USA) for 30 min and analyzed using a BD FACS Aria III Flow cytometer (Becton, Dickinson and Company, Franklin Lakes, NJ, USA). An IgG binding index, calculated as the ratio of the median AF647 fluorescence of green fluorescent protein–positive cells to that of green fluorescent protein–negative cells, of ≥2.5 was considered positive [3].

### 2.5. Statistical Analysis

Statistical analyses were performed using Prism software version 8.0 (GraphPad Software, La Jolla, CA, USA) and MedCalc version 19.0.7 (MedCalc Software Ltd., Flanders, Belgium). All values are expressed as mean ± standard error of the mean. The Mann–Whitney *U* test was used to determine the statistical significance of the fluorescence intensity. Clinical scores and categorical variables were analyzed using the analysis of variance (ANOVA) and Mann–Whitney nonparametric tests. Correlation analysis was used to evaluate the relationship of plasma LCN2 levels with MOG-IgG titers in patients with optic neuritis. Receiver operating characteristic (ROC) curves were constructed to assess and compare the ability of plasma LCN2 levels and MOG-IgG titers for predicting optic neuritis recurrence. A *p* value < 0.05 was considered statistically significant.

## 3. Results

### 3.1. Plasma LCN2 Levels and Their Relationship with MOG-IgG Titers

**Figure 1:**
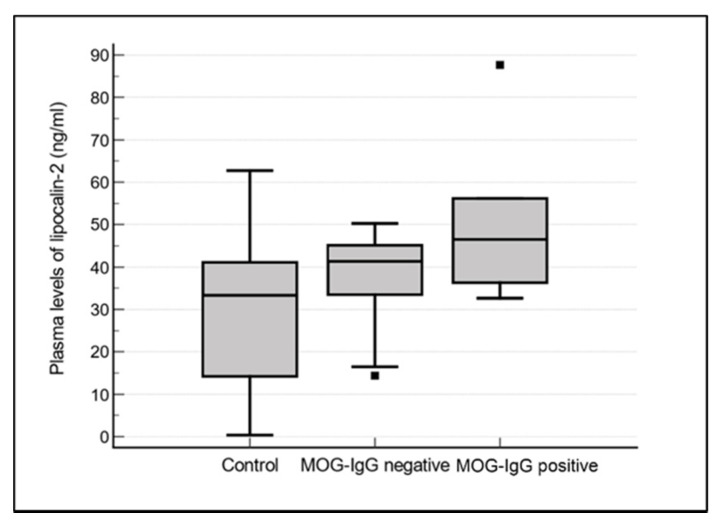
Increased plasma LCN2 levels in patients with MOG-IgG positive optic neuritis.

**Figure 2:**
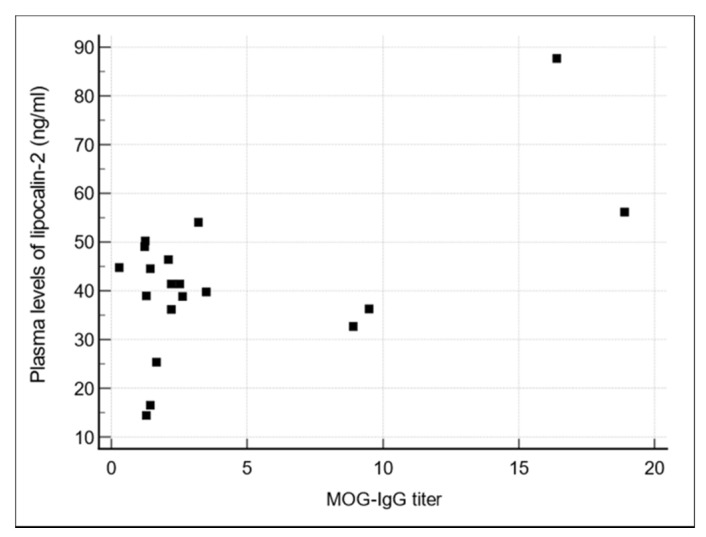
Plasma LCN2 levels were positively correlated with MOG-IgG titers.

Table 1 shows the demographic and clinical characteristics of the patients with optic neuritis and healthy controls. Among the 19 patients with optic neuritis, 6 demonstrated MOG-IgG seropositivity; the remaining 13 patients were diagnosed with MOG-IgG–negative optic neuritis. The average duration of follow-up of all the patients included in this study was 23.9 months, 25.2 months in patients with MOG-IgG positive optic neuritis, and 23.3 months in patients with MOG-IgG negative optic neuritis. Patients with MOG-IgG–positive optic neuritis had significantly higher mean plasma LCN2 levels than controls and patients with MOG-IgG–negative optic neuritis (50.96, 30.86, and 37.60 ng/mL, respectively, *p* = 0.037; Figure 1). Plasma LCN2 levels were positively correlated with MOG-IgG titers in patients with optic neuritis (*r* = 0.553, *p* = 0.0141); that is, patients with high plasma LCN2 levels demonstrated high MOG-IgG titers (Figure 2).

Quantification of plasma LCN2 levels by ELISA in healthy controls (*n* = 20), patients with MOG-IgG negative optic neuritis (*n* = 13), and patients with MOG-IgG positive optic neuritis (*n* = 6). The mean plasma LCN2 values were significantly higher in the patients with MOG-IgG positive optic neuritis when compared to controls and patients with MOG-IgG negative optic neuritis. (50.96 vs. 30.86 vs. 37.60 ng/mL, respectively, *p* = 0.037, ANOVA test) LCN2; lipocalin-2, MOG; myelin oligodendrocyte glycoprotein.

The plasma LCN2 levels were positively correlated with MOG-IgG titers in patients with optic neuritis (*r* = 0.553, *p* = 0.0141); i.e., patients with higher plasma LCN2 levels demonstrated higher titer of MOG-IgG. LCN2; lipocalin-2, MOG; myelin oligodendrocyte glycoprotein.

### 3.2. Ability to Predict Optic Neuritis Recurrence

Among the 19 patients with optic neuritis, 8 experienced optic neuritis recurrence during the follow-up period. Among the patients with recurrence, 3 demonstrated MOG-IgG seropositivity. ROC curve analysis revealed that a plasma LCN2 level of 36.32 ng/mL measured using ELISA could predict optic neuritis recurrence with a sensitivity and specificity of 62.5% and 81.8%, respectively, and a MOG-IgG titer of 3.5 measured using cell-based assays could predict optic neuritis recurrence with a sensitivity and specificity of 44.4% and 90.9%, respectively. The areas under the ROC curves (AUC) of plasma LCN2 level and MOG-IgG titer were 0.693 (95% confidence interval [CI], 0.443–0.880, *p* = 0.133) and 0.641 (95% CI, 0.400–0.840, *p* = 0.298), respectively. However, there was no significant difference between these two AUCs (95% CI, −0.266–0.448, *p* = 0.618).

## 4. Discussion

In this study, we investigated the correlation between plasma LCN2 levels and MOG-IgG titers in patients with optic neuritis. Plasma LCN2 levels were significantly higher in patients with MOG-IgG–positive optic neuritis than in controls and patients with MOG-IgG–negative optic neuritis. Additionally, plasma LCN2 levels were positively correlated with MOG-IgG titers in patients with optic neuritis. The LCN2 and MOG-IgG correlation supports the immune and inflammatory nature of MOG-IgG positive optic neuritis, as LCN2 has been strongly associated with neuroinflammation. Therefore, our results highlight the diagnostic value of plasma LCN2 levels for differentiating MOG-IgG–positive optic neuritis from MOG-IgG–negative optic neuritis.

Our results are consistent with those from our previous study, in which we found that LCN2 expression in the optic nerve was highly upregulated after EAON induction with MOG immunization and that LCN2 deficiency attenuated the pathogenesis of EAON [12]. LCN2 is now considered to play an important role in the recruitment of peripheral immune and inflammatory cells into the optic nerve to establish a proinflammatory environment [12]. Nam et al. previously reported that LCN2 administration resulted in an increased expression of interleukin-17 and interferon-gamma in MOG-specific T cells in vitro with upregulated expression of proinflammatory cytokines, chemokines, and neurotoxic molecules, such as matrix metalloproteinase-9 (MMP-9) in glial cells [16]. MMP-9 upregulation occurs in inflammatory demyelinating diseases in the CNS, and its production has been associated with blood–brain barrier damage in an experimental autoimmune encephalitis model [20]. Additionally, interaction between MMP-9 and LCN2 has been thought to enhance the damaging effect of MMP-9 on the blood–brain barrier [21]. These results may explain why optic nerve head swelling is frequently observed in patients with MOG-IgG–positive optic neuritis [3,4,11]. Our results also correspond with those of Chen et al., who reported that orbital magnetic resonance imaging of all patients with MOG-IgG–positive optic neuritis showed prominent optic nerve enhancement, and 50% of those patients demonstrated perineural enhancement with extension of enhancement to the surrounding orbital tissues during an optic neuritis attack [3].

Moreover, patients with MOG-IgG–positive optic neuritis experience more frequent recurrences with shorter durations between attacks than MOG-IgG–negative optic neuritis [3,4,11]. Thus, early identification of MOG-IgG positivity can help clinicians reduce the risk of recurrence in their patients through close monitoring for signs of recurrence and long-term prophylactic immunosuppression. Previously, MOG-IgG titers were measured using Western blot analysis or ELISA, and there was a possibility of false positive results depending on the measurement conditions [4,5]. The accuracy of MOG-IgG detection has been improved through the use of cell-based assays, which have higher sensitivity and specificity than ELISA [1,4,5]. The cell-based assay technique involves the detection of high levels of natively folded human MOG protein expressed in the cell membrane. However, consensus is lacking regarding the cell-based assay approach that provides the best results. Jarius et al. analyzed MOG-IgG titers using a cell-based assay with live HEK293 cells transfected with full-length human MOG; however, others analyzed MOG-IgG titers using a cell-based assay with formalin-fixed HEK293 cells [22]. Waters et al. introduced a novel cell-based assay for the measurement of MOG-IgG1 titers that aimed to eliminate false positive results without missing true positives in patients with low titers [19]. Our results demonstrated that plasma LCN2 levels were significantly higher in patients with MOG-IgG–positive optic neuritis than in controls and patients with MOG-IgG–negative optic neuritis, and plasma LCN2 levels were positively correlated with MOG-IgG titers. In addition, measuring plasma LCN2 levels by ELISA can be completed within a day, however, measuring plasma MOG-IgG titers takes 5 to 7 days through a relatively complicated method. Therefore, our results indicate that plasma LCN2 levels can be used in clinical practice as a prompt diagnostic marker for MOG-IgG–positive optic neuritis. Furthermore, measuring both plasma LCN2 levels and MOG-IgG titers could help avoid false positive results and overcome the limitations of single-cell assays.

The limitations of this study include its retrospective nature, relatively small sample size, and short follow-up duration. Moreover, all the participants were ethnically Korean; our results may not be applicable to other populations. Finally, because the cohort was drawn from a single tertiary center, referral bias toward patients with severe and recurrent disease is anticipated. Nevertheless, limited data are available regarding the association of plasma LCN2 levels with optic neuritis. We plan to investigate the molecular mechanism underlying the increased plasma LCN2 levels in patients with MOG-IgG–positive optic neuritis with a focus on clinical factors that may influence plasma LCN2 levels in patients with MOG-IgG associated disorders.

We believe this is the first study that evaluated the plasma LCN2 levels and their correlation with MOG-IgG titers in patients with optic neuritis. We found that patients with MOG-IgG–positive optic neuritis had significantly higher plasma LCN2 levels than healthy controls and patients with MOG-IgG–negative optic neuritis. Additionally, plasma LCN2 levels were positively correlated with MOG-IgG titers in patients with optic neuritis. The LCN2 and MOG-IgG correlation supports the immune and inflammatory nature of MOG-IgG positive optic neuritis, as LCN2 has been strongly associated with neuroinflammation. Our results imply that measurement of plasma LCN2 levels may be helpful in differentiating MOG-IgG–positive optic neuritis from MOG-IgG–negative optic neuritis. In addition, measuring both plasma LCN2 levels and MOG-IgG titers could be useful for avoiding false positive results and overcoming the limitations of single-cell assays.

## Figures and Tables

**Table 1:** Demographics and clinical characteristics of patients with optic neuritis and healthy controls.

	Patients with MOG-IgG Positive Optic Neuritis	Patients with MOG-IgG Negative Optic Neuritis	Controls
Number	6	13	20
Sex (male: female)	1:5	3:10	5:15
Age (years, mean ± SD)	27.2 ± 18.2	39.38 ± 16.4	36.3 ± 12.8
Mean visual acuity at diagnosis (LogMAR, mean ± SD)	1.08 ± 0.89	0.87 ± 0.62	NA
Mean final visual acuity (LogMAR, mean ± SD)	0.04 ± 0.08	0.42 ± 0.72	NA
Recurrence (number, %)	3 (50.0%)	5 (38.5%)	NA
Mean duration of follow-up (months, range)	25.2 (12~48)	23.3 (12~48)	NA

## Data Availability

Data are available upon request to corresponding author.
